# 3-(3-Fluoro­benz­yl)isochroman-1-one

**DOI:** 10.1107/S1600536809003304

**Published:** 2009-01-31

**Authors:** Tariq Mahmood Babar, Ghulam Qadeer, Nasim Hasan Rama, Javeed Akhtar, Madeleine Helliwell

**Affiliations:** a Department of Chemistry, Quaid-i-Azam Univeristy, Islamabad 45320, Pakistan; b Manchester Materials Science Centre and Department of Chemistry, University of Manchester, Oxford Road, Manchester M13 9PL, England

## Abstract

In the mol­ecule of the title compound, C_16_H_13_FO_2_, the aromatic rings are oriented at a dihedral angle of 74.46 (4)°. The heterocyclic ring adopts a twisted conformation. In the crystal structure, there is a weak C—H⋯π inter­action.

## Related literature

For related structures, see: Schmalle *et al.* (1982 ▶); Schnebel *et al.* (2003 ▶). For bond-length data, see: Allen *et al.* (1987 ▶). For ring-puckering parameters, see: Cremer & Pople (1975 ▶). For details of the Cambridge Structural Database, see: Allen (2002 ▶).
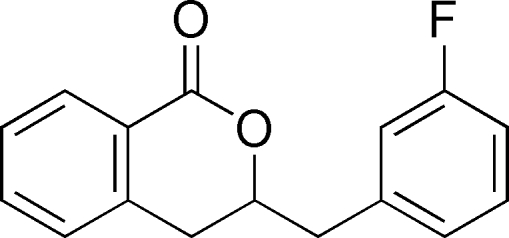


## Experimental

### 

#### Crystal data

C_16_H_13_FO_2_
*M* *_r_* = 256.26
Monoclinic, 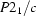

*a* = 12.6154 (16) Å
*b* = 7.6918 (10) Å
*c* = 13.0532 (17) Å
β = 103.705 (2)°
*V* = 1230.6 (3) Å^3^
*Z* = 4
Mo *K*α radiation
μ = 0.10 mm^−1^
*T* = 100 (2) K
0.50 × 0.40 × 0.10 mm

#### Data collection

Bruker SMART CCD area-detector diffractometer
Absorption correction: none
6739 measured reflections
2501 independent reflections
1805 reflections with *I* > 2σ(*I*)
*R*_int_ = 0.055

#### Refinement

*R*[*F* ^2^ > 2σ(*F* ^2^)] = 0.042
*wR*(*F* ^2^) = 0.096
*S* = 0.93
2501 reflections
172 parameters
H-atom parameters constrained
Δρ_max_ = 0.24 e Å^−3^
Δρ_min_ = −0.19 e Å^−3^

### 

Data collection: *SMART* (Bruker, 2001 ▶); cell refinement: *SAINT* (Bruker, 2002 ▶); data reduction: *SAINT*; program(s) used to solve structure: *SHELXS97* (Sheldrick, 2008 ▶); program(s) used to refine structure: *SHELXL97* (Sheldrick, 2008 ▶); molecular graphics: *SHELXTL* (Sheldrick, 2008 ▶); software used to prepare material for publication: *SHELXTL*.

## Supplementary Material

Crystal structure: contains datablocks I, global. DOI: 10.1107/S1600536809003304/hk2616sup1.cif

Structure factors: contains datablocks I. DOI: 10.1107/S1600536809003304/hk2616Isup2.hkl

Additional supplementary materials:  crystallographic information; 3D view; checkCIF report

## Figures and Tables

**Table 1:** Hydrogen-bond geometry (Å, °) *Cg*1 is the centroid of the C3–C8 ring.

*D*—H⋯*A*	*D*—H	H⋯*A*	*D*⋯*A*	*D*—H⋯*A*
C12—H12⋯*Cg*1^i^	0.95	2.95	3.806 (3)	151

Symmetry code: (i) 
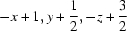
.

**Fig. 1.**
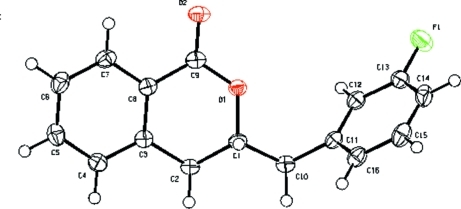
The molecular structure of the title molecule, with the atom-numbering scheme. Displacement ellipsoids are drawn at the 50% probability level.

**Fig. 2.**
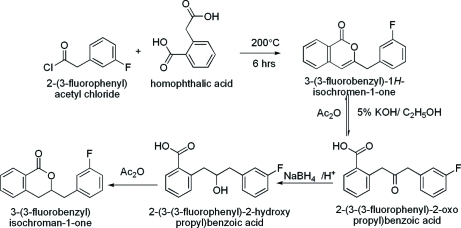
The formation of the title compound.

## supplementary crystallographic information

### Comment

The title compound was prepared in order to evalute its potential as antibacterial and antifungal agents. The CCDC search (Allen, 2002) showed that the crystal structures of rac-*exo*-tricarbonyl-(h6-3-phenyl isochromanone)chromium (Schnebel *et al.*, 2003) and 3,4-dihydro-8-hydroxy-3-(4-hydroxyphenyl)isocoumarin (Schmalle *et al.*, 1982) have been reported, which have close resemblance as far as isochromane and attached phenyl ring is considered. We report herein the synthesis and crystal structure of the title compound.

In the molecule of the title compound (Fig. 1), the bond lengths (Allen *et al.*, 1987) and angles are within normal ranges. Rings A (C3–C8) and C (C11–C16) are, of course, planar, and they are oriented at a dihedral angle of 74.46 (4)°. Ring B (O1/C1–C3/C8/C9) is not planar, having total puckering amplitude, Q_T_, of 0.467 (3) Å and twisted conformation [φ = -105.12 (3)° and θ = 118.02 (3)°] (Cremer & Pople, 1975).

In the crystal structure, there is a C—H···π interaction (Table 1).

### Experimental

As shown in Scheme 2, a mixture of homophthalic acid (1.98 g, 11.0 mmol) and 2-(3-fluorophenyl) acetyl chloride (7.91 g, 46 mmol) was heated under reflux at 473 K. After concentration, the residue was chromatographed on silica gel column using petroleum ether (333–353 K) to give 3-(3-fluorobenzyl)-1*H*-isochromen-1-one. 2-(3-(3-fluorophenyl)-2-oxopropyl)benzoic acid was obtained by refluxing a solution of 3-(3-fluorobenzyl)-1*H*-isochromen-1-one (3.6 g, 15.9 mmol) in ethanol (200 ml) and potassium hydroxide (5%,200 ml) for 6 h. NaBH4 (1.6 g) was added to a solution of 2-(3-(3-fluorophenyl)-2-oxopropyl)benzoic acid (4.23 g, 17.8 mmol) in sodium hydroxide (1%, 180 ml), and the resulting solution was stirred overnight at room temperature. After being acidified with HCl, the whole mixture was extracted with dichloromethane (2 × 15 ml). Usual work-up gave crude racemic hydroxy-acid, 2-(3-(3-fluorophenyl)-2-hydroxy propyl)benzoic acid, which was dissolved in acetic anhydride (5 ml) and heated under reflux for 2 h to get the title compound (yield; 81%, m.p, 374–375 °). The crude compound was purified by column chromatography on silica gel with petroleum ether and recrystallized in ethanol.

### Refinement

H atoms were positioned geometrically, with C—H = 0.95, 1.00 and 0.99 Å for aromatic, methine and methylene H, respectively, and constrained to ride on their parent atoms, with U_iso_(H) = 1.2U_eq_(C).

### Crystal data

**Table:**

C_16_H_13_FO_2_	*F*(000) = 536
*M**_r_* = 256.26	*D*_x_ = 1.383 Mg m^−^^3^
Monoclinic, *P*2_1_/*c*	Melting point: 374(1) K
Hall symbol: -P 2ybc	Mo *K*α radiation, λ = 0.71073 Å
*a* = 12.6154 (16) Å	Cell parameters from 1804 reflections
*b* = 7.6918 (10) Å	θ = 3.2–26.2°
*c* = 13.0532 (17) Å	µ = 0.10 mm^−^^1^
β = 103.705 (2)°	*T* = 100 K
*V* = 1230.6 (3) Å^3^	Plate, colourless
*Z* = 4	0.50 × 0.40 × 0.10 mm

### Data collection

**Table:**

Bruker SMART CCD area-detector diffractometer	1805 reflections with *I* > 2σ(*I*)
Radiation source: fine-focus sealed tube	*R*_int_ = 0.055
graphite	θ_max_ = 26.4°, θ_min_ = 1.7°
φ and ω scans	*h* = −15→15
6739 measured reflections	*k* = −8→9
2501 independent reflections	*l* = −16→9

### Refinement

**Table:**

Refinement on *F*^2^	Primary atom site location: structure-invariant direct methods
Least-squares matrix: full	Secondary atom site location: difference Fourier map
*R*[*F*^2^ > 2σ(*F*^2^)] = 0.042	Hydrogen site location: inferred from neighbouring sites
*wR*(*F*^2^) = 0.096	H-atom parameters constrained
*S* = 0.93	*w* = 1/[σ^2^(*F*_o_^2^) + (0.0463*P*)^2^] where *P* = (*F*_o_^2^ + 2*F*_c_^2^)/3
2501 reflections	(Δ/σ)_max_ < 0.001
172 parameters	Δρ_max_ = 0.24 e Å^−^^3^
0 restraints	Δρ_min_ = −0.19 e Å^−^^3^

### Special details

**Table:**

Geometry. All e.s.d.'s (except the e.s.d. in the dihedral angle between two l.s. planes) are estimated using the full covariance matrix. The cell e.s.d.'s are taken into account individually in the estimation of e.s.d.'s in distances, angles and torsion angles; correlations between e.s.d.'s in cell parameters are only used when they are defined by crystal symmetry. An approximate (isotropic) treatment of cell e.s.d.'s is used for estimating e.s.d.'s involving l.s. planes.
Refinement. Refinement of *F*^2^ against ALL reflections. The weighted *R*-factor *wR* and goodness of fit *S* are based on *F*^2^, conventional *R*-factors *R* are based on *F*, with *F* set to zero for negative *F*^2^. The threshold expression of *F*^2^ > σ(*F*^2^) is used only for calculating *R*-factors(gt) *etc*. and is not relevant to the choice of reflections for refinement. *R*-factors based on *F*^2^ are statistically about twice as large as those based on *F*, and *R*- factors based on ALL data will be even larger.

### Fractional atomic coordinates and isotropic or equivalent isotropic displacement parameters (Å^2^)

**Table:**

	*x*	*y*	*z*	*U*_iso_*/*U*_eq_	
F1	0.04488 (8)	0.11657 (13)	0.39583 (8)	0.0386 (3)	
O1	0.45079 (8)	0.20180 (13)	0.44056 (8)	0.0218 (3)	
O2	0.50042 (9)	0.34257 (14)	0.59049 (9)	0.0268 (3)	
C1	0.47167 (12)	0.1662 (2)	0.33687 (12)	0.0196 (4)	
H1	0.4666	0.2776	0.2964	0.024*	
C2	0.58453 (12)	0.0910 (2)	0.34910 (13)	0.0212 (4)	
H2A	0.5877	−0.0258	0.3815	0.025*	
H2B	0.6005	0.0783	0.2788	0.025*	
C3	0.66870 (12)	0.20666 (19)	0.41701 (12)	0.0200 (4)	
C4	0.77528 (12)	0.2189 (2)	0.40595 (13)	0.0241 (4)	
H4	0.7972	0.1532	0.3529	0.029*	
C5	0.84964 (13)	0.3259 (2)	0.47153 (14)	0.0288 (4)	
H5	0.9225	0.3325	0.4636	0.035*	
C6	0.81851 (13)	0.4239 (2)	0.54894 (14)	0.0295 (4)	
H6	0.8699	0.4974	0.5938	0.035*	
C7	0.71298 (13)	0.4138 (2)	0.56045 (14)	0.0259 (4)	
H7	0.6915	0.4811	0.6131	0.031*	
C8	0.63765 (12)	0.30552 (19)	0.49515 (13)	0.0206 (4)	
C9	0.52632 (13)	0.28886 (19)	0.51240 (13)	0.0214 (4)	
C10	0.38215 (12)	0.0446 (2)	0.28054 (13)	0.0219 (4)	
H10A	0.3948	0.0162	0.2104	0.026*	
H10B	0.3872	−0.0651	0.3211	0.026*	
C11	0.26821 (12)	0.1165 (2)	0.26575 (13)	0.0209 (4)	
C12	0.20723 (12)	0.0833 (2)	0.33946 (13)	0.0230 (4)	
H12	0.2368	0.0155	0.4003	0.028*	
C13	0.10394 (13)	0.1499 (2)	0.32292 (14)	0.0256 (4)	
C14	0.05618 (14)	0.2488 (2)	0.23648 (15)	0.0301 (4)	
H14	−0.0159	0.2923	0.2272	0.036*	
C15	0.11730 (13)	0.2824 (2)	0.16351 (15)	0.0310 (4)	
H15	0.0871	0.3507	0.1030	0.037*	
C16	0.22210 (13)	0.2173 (2)	0.17798 (14)	0.0263 (4)	
H16	0.2631	0.2417	0.1273	0.032*	

### Atomic displacement parameters (Å^2^)

**Table:**

	*U*^11^	*U*^22^	*U*^33^	*U*^12^	*U*^13^	*U*^23^
F1	0.0329 (6)	0.0451 (7)	0.0432 (7)	−0.0007 (5)	0.0195 (5)	−0.0039 (5)
O1	0.0239 (6)	0.0238 (6)	0.0181 (6)	0.0009 (5)	0.0059 (5)	−0.0008 (5)
O2	0.0334 (7)	0.0281 (6)	0.0203 (7)	0.0060 (5)	0.0091 (6)	−0.0012 (5)
C1	0.0257 (8)	0.0187 (8)	0.0150 (8)	0.0008 (6)	0.0058 (7)	0.0018 (7)
C2	0.0258 (9)	0.0201 (8)	0.0189 (9)	0.0005 (7)	0.0079 (7)	−0.0004 (7)
C3	0.0242 (8)	0.0161 (8)	0.0189 (9)	0.0045 (6)	0.0038 (7)	0.0046 (7)
C4	0.0251 (9)	0.0212 (8)	0.0262 (10)	0.0028 (7)	0.0065 (8)	0.0008 (7)
C5	0.0221 (9)	0.0258 (9)	0.0380 (11)	0.0020 (7)	0.0063 (8)	0.0015 (8)
C6	0.0271 (9)	0.0229 (9)	0.0341 (11)	−0.0002 (7)	−0.0016 (8)	−0.0044 (8)
C7	0.0311 (9)	0.0210 (9)	0.0236 (10)	0.0053 (7)	0.0028 (8)	−0.0020 (7)
C8	0.0241 (8)	0.0175 (8)	0.0191 (9)	0.0043 (6)	0.0028 (7)	0.0029 (7)
C9	0.0286 (9)	0.0163 (8)	0.0190 (9)	0.0046 (7)	0.0052 (8)	0.0041 (7)
C10	0.0263 (9)	0.0183 (8)	0.0218 (9)	−0.0005 (6)	0.0070 (8)	0.0004 (7)
C11	0.0238 (9)	0.0157 (8)	0.0220 (9)	−0.0038 (6)	0.0031 (7)	−0.0029 (7)
C12	0.0255 (9)	0.0198 (8)	0.0221 (9)	−0.0009 (7)	0.0028 (8)	−0.0002 (7)
C13	0.0248 (9)	0.0247 (9)	0.0293 (10)	−0.0048 (7)	0.0100 (8)	−0.0063 (8)
C14	0.0219 (9)	0.0236 (9)	0.0409 (12)	0.0016 (7)	−0.0002 (8)	−0.0077 (8)
C15	0.0314 (10)	0.0248 (9)	0.0312 (11)	−0.0001 (7)	−0.0039 (9)	0.0033 (8)
C16	0.0290 (9)	0.0249 (9)	0.0232 (10)	−0.0051 (7)	0.0030 (8)	0.0013 (8)

### Geometric parameters (Å, °)

**Table:**

F1—C13	1.3648 (19)	C6—H6	0.9500
O1—C1	1.4646 (18)	C7—C8	1.393 (2)
O1—C9	1.3461 (19)	C7—H7	0.9500
O2—C9	1.2141 (19)	C8—C9	1.480 (2)
C1—C2	1.510 (2)	C10—C11	1.509 (2)
C1—C10	1.516 (2)	C10—H10A	0.9900
C1—H1	1.0000	C10—H10B	0.9900
C2—C3	1.503 (2)	C11—C12	1.391 (2)
C2—H2A	0.9900	C11—C16	1.391 (2)
C2—H2B	0.9900	C12—C13	1.369 (2)
C3—C4	1.389 (2)	C12—H12	0.9500
C3—C8	1.400 (2)	C13—C14	1.376 (3)
C4—C5	1.382 (2)	C14—C15	1.384 (2)
C4—H4	0.9500	C14—H14	0.9500
C5—C6	1.390 (2)	C15—C16	1.384 (2)
C5—H5	0.9500	C15—H15	0.9500
C6—C7	1.377 (2)	C16—H16	0.9500
			
C9—O1—C1	118.95 (12)	C7—C8—C9	119.56 (14)
O1—C1—C2	110.20 (13)	C3—C8—C9	120.21 (14)
O1—C1—C10	106.69 (12)	O2—C9—O1	117.85 (14)
C2—C1—C10	112.96 (13)	O2—C9—C8	123.52 (15)
O1—C1—H1	109.0	O1—C9—C8	118.54 (14)
C2—C1—H1	109.0	C11—C10—C1	114.41 (13)
C10—C1—H1	109.0	C11—C10—H10A	108.7
C3—C2—C1	110.62 (12)	C1—C10—H10A	108.7
C3—C2—H2A	109.5	C11—C10—H10B	108.7
C1—C2—H2A	109.5	C1—C10—H10B	108.7
C3—C2—H2B	109.5	H10A—C10—H10B	107.6
C1—C2—H2B	109.5	C12—C11—C16	118.69 (15)
H2A—C2—H2B	108.1	C12—C11—C10	120.69 (15)
C4—C3—C8	118.91 (15)	C16—C11—C10	120.61 (15)
C4—C3—C2	123.01 (14)	C13—C12—C11	118.96 (16)
C8—C3—C2	118.07 (14)	C13—C12—H12	120.5
C5—C4—C3	120.51 (15)	C11—C12—H12	120.5
C5—C4—H4	119.7	F1—C13—C12	118.44 (16)
C3—C4—H4	119.7	F1—C13—C14	118.07 (15)
C4—C5—C6	120.40 (15)	C12—C13—C14	123.49 (17)
C4—C5—H5	119.8	C13—C14—C15	117.42 (15)
C6—C5—H5	119.8	C13—C14—H14	121.3
C7—C6—C5	119.75 (16)	C15—C14—H14	121.3
C7—C6—H6	120.1	C14—C15—C16	120.54 (17)
C5—C6—H6	120.1	C14—C15—H15	119.7
C6—C7—C8	120.26 (16)	C16—C15—H15	119.7
C6—C7—H7	119.9	C15—C16—C11	120.89 (16)
C8—C7—H7	119.9	C15—C16—H16	119.6
C7—C8—C3	120.17 (15)	C11—C16—H16	119.6
			
C9—O1—C1—C2	48.49 (17)	C7—C8—C9—O2	−12.0 (2)
C9—O1—C1—C10	171.47 (12)	C3—C8—C9—O2	164.92 (15)
O1—C1—C2—C3	−53.65 (16)	C7—C8—C9—O1	171.54 (14)
C10—C1—C2—C3	−172.87 (13)	C3—C8—C9—O1	−11.5 (2)
C1—C2—C3—C4	−150.46 (15)	O1—C1—C10—C11	59.46 (17)
C1—C2—C3—C8	29.9 (2)	C2—C1—C10—C11	−179.30 (14)
C8—C3—C4—C5	0.5 (2)	C1—C10—C11—C12	−92.74 (17)
C2—C3—C4—C5	−179.19 (15)	C1—C10—C11—C16	87.32 (19)
C3—C4—C5—C6	−0.5 (2)	C16—C11—C12—C13	0.4 (2)
C4—C5—C6—C7	0.1 (3)	C10—C11—C12—C13	−179.59 (14)
C5—C6—C7—C8	0.3 (3)	C11—C12—C13—F1	−179.96 (13)
C6—C7—C8—C3	−0.3 (2)	C11—C12—C13—C14	0.1 (2)
C6—C7—C8—C9	176.63 (14)	F1—C13—C14—C15	179.63 (14)
C4—C3—C8—C7	−0.1 (2)	C12—C13—C14—C15	−0.5 (3)
C2—C3—C8—C7	179.61 (14)	C13—C14—C15—C16	0.3 (2)
C4—C3—C8—C9	−176.99 (14)	C14—C15—C16—C11	0.1 (2)
C2—C3—C8—C9	2.7 (2)	C12—C11—C16—C15	−0.5 (2)
C1—O1—C9—O2	167.83 (13)	C10—C11—C16—C15	179.45 (14)
C1—O1—C9—C8	−15.51 (19)		

### Hydrogen-bond geometry (Å, °)

**Table:**

*D*—H···*A*	*D*—H	H···*A*	*D*···*A*	*D*—H···*A*
C12—H12···Cg1^i^	0.95	2.95	3.806 (3)	151

Symmetry codes: (i) −*x*+1, *y*+1/2, −*z*+3/2.
